# Extended-Spectrum β-Lactamase-Producing *Escherichia coli* Isolated from Food-Producing Animals in Tamaulipas, Mexico

**DOI:** 10.3390/antibiotics12061010

**Published:** 2023-06-05

**Authors:** Antonio Mandujano, Diana Verónica Cortés-Espinosa, José Vásquez-Villanueva, Paulina Guel, Gildardo Rivera, Karina Juárez-Rendón, Wendy Lizeth Cruz-Pulido, Guadalupe Aguilera-Arreola, Abraham Guerrero, Virgilio Bocanegra-García, Ana Verónica Martínez-Vázquez

**Affiliations:** 1 Centro de Biotecnología Genómica, Instituto Politécnico Nacional, Tamaulipas C.P. 88710, Mexico; jamh_93@hotmail.com (A.M.); gguelg1700@alumno.ipn.mx (P.G.); gildardors@hotmail.com (G.R.); kjuarezr@ipn.mx (K.J.-R.); 2 Centro de Investigación en Biotecnología Aplicada, Instituto Politécnico Nacional, Tlaxcala C.P. 90700, Mexico; dcortes@ipn.mx; 3 Facultad de Medicina Veterinaria y Zootecnia, Universidad Autónoma de Tamaulipas, Cd. Victoria C.P. 87274, Mexico; jvazquez@docentes.uat.edu.mx; 4 Escuela de Ciencias de la Salud, Universidad del Valle de México, Reynosa C.P. 88760, Mexico; wendy.cruz@uvmnet.edu; 5 Escuela Nacional de Ciencias Biológicas, Instituto Politécnico Nacional, México City C.P. 11340, Mexico; 6 Consejo Nacional de Ciencia y Tecnología (CONACyT), Centro de Investigación en Alimentación y Desarrollo (CIAD), Mazatlán C.P. 82100, Mexico; aguerrero@ciad.mx

**Keywords:** *Escherichia coli*, antimicrobial, resistance, ESBL, livestock

## Abstract

Extended-spectrum β-lactamase (ESBL)-producing *E. coli* has become an important global problem for the public health sector. This study aims to investigate the *E. coli* antimicrobial resistance profile among living food-producing animals in Tamaulipas, Mexico. A total of 200 fecal samples were collected from bovines, pigs, chickens and sheep. A total of 5.0% of the strains were phenotypically confirmed as ESBL producers. A high percentage of phenotypic antimicrobial resistance was observed against gentamicin (93.3%), tetracycline (86.6%) and streptomycin (83.3%). The gentamicin-resistant strains showed MDR, distributed among 27 resistance patterns to different antimicrobials. The antimicrobial resistance gene *tet*(A) was detected in 73.3% of isolates, *aad*A1 in 60.0% and *sul*2 in 43.3% of strains. The *bla*_CTX-M_ gene was found in 23.3% of strains. The virulence gene *hlyA* was detected in 43.3% of isolates; *stx*1 and *stx*2 were not detected in any strain. The phylotyping indicated that the isolates belonged to groups A (33.3%), B1 (16.6%), B2 (40.0%) and D (10.0%). These results show that food-producing animals might be a reservoir of ESBL-producing bacteria and may play a role in their spread.

## 1. Introduction

Antimicrobial resistance (AMR) is considered to be one of the main threats to public health worldwide [1]. In particular, the extended-spectrum β-lactamase (ESBL)-producing strains stand out due to their plasmid-encoded enzymes that are capable of providing resistance to most beta-lactam antibiotics, including penicillin, cephalosporins and monobactams. In addition, they are often resistant to other classes of antibiotics such as aminoglycosides, fluoroquinolones and trimethoprim/sulfamethoxazole, making them more difficult to treat [2,3,4]. Infections with ESBL-producing Enterobacteriaceae are associated with worse clinical outcomes, higher mortality rates, extended hospital stays and more significant expenses compared with similar infections with bacteria that do not produce ESBL [3]. Most of this problem can be attributed to the spread of ESBL-producing *Escherichia coli* and *Klebsiella pneumonia*. They are not only restricted to healthcare-associated isolates confined within clinical settings since these strains can be commonly isolated from water, soil, domestic animals and food-producing animals [5,6,7]. As a better approach to this health problem, derived from antibiotic-resistant bacteria and their transmission models to humans, the “One Health” concept was implemented. The One Health initiative aims to achieve optimal health outcomes by recognizing the interconnectedness between people, animals, plants and their shared environment [5]. Several potential sources of ESBL-producing *E. coli* (ESBL-EC) for human infection or colonization were identified, including food-producing animals [6]. Although several studies concerning ESBL strains were carried out in hospital settings, environmental reservoirs recently received attention, making it challenging to estimate their potential risk to public health and control their spread. Specifically, the isolation of ESBL-producing *E. coli* from livestock has been increasingly reported worldwide due to the continuous use of antimicrobials in livestock, promoting the emergence of multidrug-resistant pathogens [8]. For example, in Mexico, some studies reported the presence of ESBL strains in meat retailed to the public [9,10]; however, more information is needed regarding the prevalence of these strains during meat handling or in living livestock prior to slaughter. In previous studies on livestock from Mexico, a high percentage of antimicrobial resistance and MDR were reported; however, these studies did not include information concerning the presence of ESBL strains [11,12,13,14]. For this reason, the present study aims to investigate the prevalence of ESBL-producing *E. coli* (ESBL-EC) isolated from living food-producing animals and their antimicrobial resistance profiles in Tamaulipas, Mexico.

## 2. Results

### 2.1. Identification of ESBL-EC

From the 200 samples tested, 600 *E. coli* strains were isolated in our study from 50 bovines, 50 chickens, 50 pigs and 50 sheep. The samples were collected in the central region of Tamaulipas, Mexico. The overall prevalence of ESBL-EC samples was 11.5% (95%, Cl: 6.0–16.0%), and the ESBL-positive strains as a percentage of the total number of *E. coli* strains was 5.0% (95%, Cl: 2.0–7.3%); the highest percentages of the ESBL strains were observed in samples from pigs and chickens, at 7.3% and 7.3%, respectively (Table 1).

### 2.2. Antimicrobial Susceptibility

Among the 30 ESBL-EC strains that were analyzed for this study, 100% (30/30) showed resistance to at least one antibiotic tested. In contrast, 93.3% (28/30) of the isolates exhibited a multidrug-resistance phenotype (resistant to ≥3 groups of antimicrobials). In addition, 27 phenotypic resistance patterns were observed, of which 1 was repeated in four strains, and the remaining 26 were unique and different (Table 2). The multiple antibiotic resistance index (MARI) values were greater than 0.2 in all the strains. The results reveal that the highest percentages of antimicrobial resistance of the ESBL-EC strains was to gentamicin (GE) (93.3%; 28/30), tetracycline (TE) (86.6%; 26/30) and streptomycin (S) (83.3%; 25/30). In contrast, most of the strains were sensitive to nitrofurantoin (F/M) (100%; 30/30) and netilmicin (NET) (83.3%; 25/30) (Table 3). From the analysis used to detect the presence of genes related to antimicrobial resistance, we found that the *tet*A gene was the most prevalent gene in the 30 ESBL-EC strains; it was revealed that 73.3% (22/30) of the isolates had the *aad*A1 gene, and this gene determined a resistance to aminoglycosides in 60.0% (18/30) of cases (Table 3). On the other hand, the results show that 50% (15/30) of the analyzed ESBL-EC strains harbored class 1 integrons (*intl*1). Class 2 and 3 integrons (*intl*2 and *intl3*) were not detected in any of the analyzed strains.

### 2.3. Detection of Virulence Factors

All of the tested strains were negative in the analysis performed to detect the *stx*1 and *stx*2 genes. The *hyl*A gene was identified in 43.3% (13/30) of the ESBL-EC strains, in which the amplified band of 569 pb was detected; most of these were isolated from the pigs, only five were isolated from the sheep, and one was isolated from the chicken samples.

### 2.4. Phylogenetic Groups

**Figure 1:**
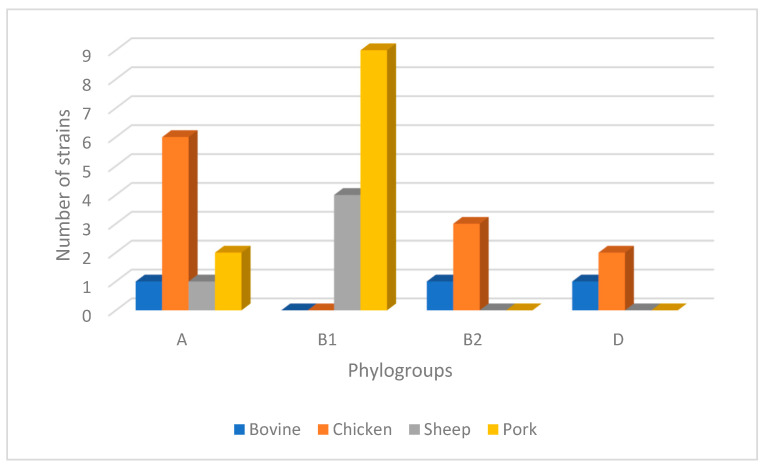
Distribution of phylogenetic groups in ESBL-EC strains isolated from livestock. The most common phylogroup of *Escherichia coli* isolates was phylogroup B2, followed by phylogroup A, phylogroup B1 and phylogroup D.

The ESBL-EC strains were distributed into the following four phylogenetic groups: A = 33.3% (10/30), B1 = 16.6% (5/30), B2 = 40% (12/30) and D = 10% (3/30) (Figure 1).

## 3. Discussion

*E. coli* is an interesting study model given its omnipresence in nature as a commensal and pathogen, being one of the primary vehicles that can transmit resistance and virulence genes between different species [15]. The monitoring of antibiotic-resistant *E. coli* strains from food animals and products is vital to determine its potential risk to humans [16]. This study found that 5.0% of strains recovered from livestock samples (cattle, pigs, chickens and sheep) were ESBL-EC. These results are similar to those reported by Benavides et al. (2021) in Chile of 3.0% (cattle, pigs, chickens, sheep and goats) [5]. However, if the prevalence of ESBL-EC is considered only in chicken samples, Sanou et al. (2022) [17] found a 7.8% incidence of ESBL-EC in chickens, similar to our results (7.3% in chicken samples). However, the percentage incidence of ESBL-EC was much higher in most other studies, reportedly between 3 and 68% [17,18,19,20,21]. An example of this includes the results published by Li et al. (2022) from China, who reported the incidence of ESBL-EC in chicken samples to be 34.3% [18]; Shafiq et al. (2022) in Pakistan, who reported 68% [19]; Giufre et al. (2021) in Italy, who reported 43.6% [20] or Sghaier et al. (2019) in Tunisia, who reported 51.6% [21]. On the other hand, considering only the pig samples in our current study, we detected a 7.3% incidence of ESBL-EC. However, in other similar studies, the prevalence was higher; for example, the results published by Sanou et al. (2022) in Africa reported an ESBL-EC incidence of 63% [17], Giufre et al. in Italy reported 27% [20] and Miltgen et al. (2022) in Reunion Island reported 28.2% [22]. The high ESBL percentages reported in some studies are not surprising, given that β-lactam antibiotics are widely used in livestock. It is necessary to consider that farm animals are frequently exposed to the use of antibiotics, such as β-lactams, therapeutically (to treat clinically sick animals), for prophylaxis (given to healthy animals at risk of infection to prevent it from occurring), for metaphylaxis (to treat diseased animals in the same group as healthy animals) and, in some countries, for growth-promoting purposes (as a feed additive) [23]. The percentages of antibiotic resistance can vary from region to region due to the legislative measures on the use of antibiotics in each region or the type of management to which the animals are subjected. Considering the comparison of the current results with similar studies in other countries, the samples included from Tamaulipas exhibited a low percentage of ESBL-EC strains. However, it is essential to remember that, although it may seem to be a low percentage, these strains can spread their antibiotic resistance to other bacteria and can represent a risk to public health if the food produced by infected livestock is improperly handled.

In addition to the antibiotics in the β-lactam group, among the antimicrobials most commonly used in animal production are the tetracyclines, phenicols (chloramphenicol) and aminoglycosides (streptomycin) [24]. Most ESBL-EC strains isolated in this study were shown to be multidrug resistant (resistant to ≥3 groups of antibiotics). This may be due to the excessive use of antibiotics in livestock that can induce and accelerate the development of resistance in bacteria. The ESBL-EC strains isolated in this study showed a high rate of co-resistance to antibiotics, such as gentamicin in 93.3% of strains (28/30), tetracycline in 86.6% of strains (26/30) and streptomycin in 83.3% of strains (25/30). This high percentage of gentamicin-resistant ESBL-EC strains may be of interest, considering that it is an alternative recommended antibiotic for the treatment of some types of ESBL infections [25]. Although carbapenem treatment is considered the “gold standard” for severe and invasive ESBL Enterobacteriaceae infections [26], a combination regimen with aminoglycosides is also used [27,28]. However, the activity of aminoglycosides against ESBL-producing Enterobacteriaceae varies according to the geographical region [28]. As far as our review goes, only two previous studies focused on resistant bacteria in livestock were published in Tamaulipas. However, the results of Martinez et al. (2021) and Vazquez et al. (2023) only include antibiotic resistance in bovines, without considering other livestock species or ESBL-EC strains. The only previous studies with ESBL-EC strains in Tamaulipas were carried out by Martinez et al. (2022) with commercial meat (beef, pork and chicken), which showed 6.5% ESBL-EC, and reference [10]. Although these percentages of antibiotic-resistant strains in meat are lower than those obtained in livestock for the current study, they cannot be considered entirely comparable. It should be noted that although the meat samples were for sale in Tamaulipas, not all originated from local livestock production, since some businesses bring the meat from other regions. However, the discussion regarding the presence of antibiotic-resistant strains in Tamaulipas, whether or not they originated from local livestock, is relevant, since they play an essential role in the distribution of antibiotic resistance genes (ARG). At the same time, ARGs with a food or animal origin can be transferred to other bacteria via horizontal gene transfer (HGT), which plays a key role in acquiring, accumulating and disseminating ARGs to the most virulent bacteria [23,29] that can cause infection in humans.

The health risk associated with the spread of antibiotic resistance in the environment is estimated using the multiple antibiotic resistance index (MARI) [16,30]. In this study, all of the values obtained show that the MARI was greater than 0.2, suggesting that the ESBL-EC strains originated from an environment with a high contamination or overuse of antibiotics. The resistance genes detected in the ESBL-EC isolates via PCR revealed that the CTX-M gene was the most prevalent β-lactam gene (23.3%; 7/30), which corresponds to the phenotypic resistance to the third and fourth generation cephalosporins. Usually, *bla*_CTX-M_ genes are located on transferable plasmids, which could spread among animal, environmental and human *E. coli* isolates [31]. Regarding the genes associated with resistance to non-β-lactam antibiotics, *tet*A was the most prevalent gene in the ESBL-EC strains. The high percentage of phenotypic resistance to tetracycline (86.6%) and genotypic detection (*tet*A 73.3% and *tet*B 13.3%) in these ESBL-EC strains is not surprising, since it is an antibiotic that is widely used in animal and human infections due to its wide availability and low cost. On the other hand, although quinolones and β-lactams are widely used worldwide for the treatment of many infectious diseases [32], none of the ESBL strains contained the *qnr*A gene, and only 20% had the *qnr*B gene. The *qnr* genes are usually integrated multimers, and plasmids often harbor other antibiotic resistance genes such as ESBLs, favoring their selection and dissemination [32,33]. This is considered to be a promoter of the spread of multidrug resistance. In the resistance capacity to antibiotics developed by some bacteria, integrons play an important role. They are the genetic platform enabling the bacteria to capture, store and reorder antibiotic resistance cassettes through site-specific recombination, facilitating their dissemination [34]. This study shows that 93.3% (28/30) of the ESBL-EC strains were MDR; of these, 50% (15/30) contained class 1 integrons, indicating that they were strains capable of dispersing antibiotic resistance. The class-1 integron-positive isolates were resistant to aminoglycosides (*aad*A1 14/15, *str*B 8/15), tetracyclines (*tet*A 15/15) and sulfonamides (*sul*1 8/15, *sul*2 10/15). Integrons, as mobile elements, can transmit the resistance genes from one organism to another; this represents an extremely important challenge in livestock since the performance of infection control programs is poor [35].

To determine the potential pathogenicity of the strains, we used the phylogenetic characterization system developed by Clermont et al. [35] to classify the strains into four phylogroups (A, B1, B2 and D). The strains belonging to the various phylogroups differ in their genome size, variable gene content, disease association, ecological niche and life history characteristics [36]. For this reason, the use of the phylogroup classification was employed in the study of ecological niches and lifestyles in bacterial pathogens, and improves our understanding of the population structure, providing invaluable epidemiological information [37]. Studies have shown that strains associated with virulent extraintestinal infection usually belong to phylogeny groups B2 or D, and that the commensal *E. coli* isolates are generally affiliated with groups A and B1 [38,39,40,41].

In this study, the ESBL-EC strains were classified as follows: 33.3% (10/30) were classified as phylogroup A, which is strongly associated with human sources [37]. A further 16.6% (5/30) were classified as phylogroup B1, which has a significant relationship with food sources [37]. Moreover, 40% (12/30) of the strains were in phylogroup B2, which is associated with herbivorous and omnivorous mammals, and is considered the leading cause of extraintestinal infections in humans and diarrheal diseases [37]. Finally, 10% (3/30) of the strains were classified as phylogroup D, which is also related to human sources [37]. Furthermore, it is interesting to note that in phylogroup B, 66.6% (8/12) of the strains had class 1 integrons, and 50% (6/12) had the *hly*A gene.

To our knowledge, this is the first study on ESBL-EC strains in livestock from northeast Mexico (Tamaulipas state). On the other hand, the samples are obtained only from the central zone of the state, which could be considered a limitation, since the findings cannot be generalized to the entire state. However, being the first data generated in the state, they serve as an indicator of the current situation and a basis for continuing with studies of this type.

In general, the ESBL-EC strains were identified in the four livestock species analyzed; although it may seem to be a low percentage (5.0%), the presence of these strains always represents a focus of alert due to their role in the spread of the resistance to antibiotics. Five strains isolated from pigs simultaneously presented a class 1 integron and the *hly*A gene, and all belonged to the B2 phylogroup and were MDR; therefore, they stand out as a potential risk to public health. These results highlight the importance of carrying out this type of surveillance study in livestock to determine the current situation of bacterial resistance, the virulence factors present and the risk they represent to the population.

## 4. Materials and Methods

### 4.1. Identification of ESBL-EC

The study was conducted between January and September 2021 in the central region of the state of Tamaulipas, Mexico. Fecal samples were collected from adult animals, including cattle, chickens, pigs and sheep. Bovine and sheep samples were acquired via rectal retrieval using disposable gloves, while chicken and pig samples were recovered from cloacal swabs. All samples collected were aseptically manipulated, labelled and stored individually for transport to the laboratory. To isolate *E. coli*, each sample was inoculated into lactose broth (BD Difco™), homogenized and incubated at 37 °C for 24 h. Subsequently, one loop of the culture was streaked onto eosin–methylene blue (EMB) agar (BD Becton Dickinson and Co., Mexico) plates and incubated at 37 °C for 18–24 h. After incubation, three presumptive colonies with characteristics corresponding to *E. coli* morphology were randomly selected. Each colony was transferred to tryptic soy agar (TSA) (BD Becton Dickinson and Co) plates and incubated for 24 h at 37 °C to obtain a pure culture. Standard biochemical tests were applied to confirm the identity of the *E. coli*, including methyl red, Voges–Proskauer, lactose and sugar fermentation, indole and motility production and citrate metabolism. The identification was made via MALDI-TOF mass spectrophotometry and PCR. The DNA was obtained from a pure culture on tryptic soy agar (BD Becton Dickinson and Co., Cuautitlán Izcalli, Mexico) via lysis of a bacterial cell suspension at 95 °C for 15 min, followed by centrifugation at 13,000× *g* for 3 min. The *mdh* gene was employed to confirm the identification of *E. coli* as described by Vasquez et al. [42]. All strains that were confirmed to be *E. coli* were subjected to the phenotypic screening of ESBL production using the double-disk synergy test according to the European Committee on Antimicrobial Susceptibility Testing guidelines [43].

### 4.2. Antimicrobial Susceptibility

Antimicrobial susceptibility testing was applied to ESBL-EC strains with 17 antimicrobials from eight classes by the standard Kirby–Bauer disk diffusion method according to guidelines of the Clinical and Laboratory Standards Institute [44]. The antimicrobials used were streptomycin (S, 10 µg), gentamicin (GE, 30 μg), netilmicin (NET, 30 μg), amikacin (AN, 30 μg), chloramphenicol (C, 30 μg), ampicillin (AM, 10 μg), cephalothin (CF, 30 μg), cefepime (FEP, 30 μg), cefotaxime (CTX, 30 μg), ceftazidime (CAZ, 30 μg), ceftriaxone (CRO, 30 μg), amoxicillin–clavulanate (30 μg), ciprofloxacin (CIP, 5 μg), trimethoprim–sulfamethoxazole (STX, 25 μg), tetracycline (T, 30 μg), levofloxacin (LV, 30 μg) and nitrofurantoin (F/M, 100 μg). The results were interpreted in accordance with the CLSI M100-Ed31 recommendations [45]. *E. coli* ATCC 25,922 and ATCC 3518 were used as the control strains. The results were used to calculate the Antibiotic Resistance Index (ARI) and Multiple Antibiotic Resistance (MAR) index for the bacteria according to Jalil et al. [16]. Strains showing a MAR index above 0.2 indicate high resistance in that specific isolate [16].

All ESBL-EC isolates were analyzed to detect the presence of twelve resistance genes associated with resistance to β-lactamases (*bla*_CTX-M_*, bla*_CTX-M2_*, bla*_CTX-M3_, *bla*_TEM_, *bla*_SHV_), tetracyclines (*tet*A and *tet*B), streptomycin (*str*A and *str*B), aminoglycosides (*aac(3)*-VI and *aad*A1), sulfonamides (*sul*1, *sul*2 and *sul*3) and quinolones (*qnr*A and *qnr*B) [46,47,48]. The presence of the *intI*1, *intI*2 and *intI*3 genes (encoding for class 1, class 2 and class 3) was determined via polymerase chain reaction (PCR) [49]. Negative controls (samples without a DNA template) and positive controls (samples with DNA from the collection of the Instituto Politécnico Nacional) were included in all PCR assays. The results were visualized via electrophoresis in 2.0% agarose gel at 100 V for 45 min. A molecular marker was run concurrently (100 pb Promega).

### 4.3. Detection of Virulence Factors

PCR assay targeted three virulence factors, Shiga toxin-producing *E. coli* (*stx*1 and *stx*2) and extraintestinal pathogenic *E. coli*-associated toxin (*hyl*A), under the conditions described by Canizalez et al. [50].

### 4.4. Phylogenetic Groups

ESBL-producing isolates were classified in phylogenetic groups by amplifying fragments of the *chuA* (279 bp) and *yjaA* (211 bp) genes, and the DNA fragment TspE4.C2 (152 bp) as described by Clermont et al. [16]. Briefly, groups were assigned on the basis of different combinations of the presence and/or absence of the three amplicons (A, B1, B2 and D).

## 5. Conclusions

The presence of ESBL-EC strains in livestock used for food production warrants a focus of attention on the problem of bacterial resistance. This is particularly important as our study found that a high percentage of the tested ESBL-EC strains in livestock used for food production were MDR. In Mexico and in the northeast of the country, there have been few studies that address this issue in animals. Consequently, there is no thorough understanding of the potential transmission of these strains. An interesting point to highlight from the results of this study is the presence of ESBL-EC strains in combination with the class 1 integron and *hly*A gene, and its classification within the B2 phylogroup (considered pathogenic). This may represent a potential risk to public health and a niche method of distribution of ARGs to other bacterial strains. Results such as these indicate the importance of monitoring bacterial resistance and its reservoirs, which contribute to a One Health vision that helps us to gain a better understanding of the transmission of bacterial resistance.

## Figures and Tables

**Table 1:** Prevalence of ESBL-producing *E. coli* in livestock.

Type of Sample	Number of Samples	ESBL-Positive Samples	Number of Strains of *E. coli*	CTX-Resistant or Intermediate	ESBL-Positive Strains
Bovines	50	6.0% (3/50)	150	36.6% (55/150)	2.0% (3/150)
Chickens	50	16.0% (8/50)	150	35.3% (53/150)	7.3% (11/150)
Pigs	50	10.0% (5/50)	150	37.3% (56/150)	7.3% (11/150)
Sheep	50	14.0% (7/50)	150	44.6% (67/150)	3.3% (5/150)
Total	200	11.5% (23/200)	600	38.5% (231/600)	5.0% (30/600)

**Table 2:** Resistance pattern and MARI in ESBL-EC strains isolated from livestock.

Sample	No. of Isolates	Resistance Patterns	No. of Antibiotics	MARI
Bovines	1	GE-NET-S-C-AM-FEP-STX-TE	8	0.471
	2	AN-GE-NET-S-CF-FEP-CTX-TE	8	0.471
	3	AN-GE-NET-S-CTX-CRO-CIP-TE	8	0.471
Chickens	1	AN-GE-CF-CTX-CRO	5	0.294
	2	AN-GE-CTX-CRO-TE	5	0.294
	3	S-C-FEP-CIP-STX-TE-LV	7	0.412
	4	AN-GE-C-CTX-CRO-CIP-STX-TE-LV	9	0.529
	5	AN-GE-NET-S-C-AM-CRO-STX-TE	9	0.529
	6	AN-GE-S-CF-FEP-CTX-CRO-AmC-TE	9	0.529
	7	AN-GE-S-CF-CTX-CRO-AmC-STX-TE	9	0.529
	8	AN-GE-AM-CF-CTX-CRO-AmC-STX-TE	9	0.529
	9	AN-GE-S-C-AM-FEP-CTX-CAZ-CRO-STX	10	0.588
	10	AN-G-CF-CTX-CRO-AmC-CIP-STX-TE-LV	10	0.588
	11	AN-GE-S-C-CF-FEP-CTX-CRO-CIP-STX-TE-LV	12	0.706
Sheep	1	GE-S-AM-CF-TE	5	0.294
	2	AN-GE-S-C-AM	5	0.294
	3	GE-S-CF-CTX-TE	5	0.294
	4	AN-GE-S-AM-CF-CTX	6	0.353
	5	GE-S-C-AM-CF-CTX-TE	7	0.412
Pigs	1	AN-GE-S-C-AM-CF-AmC-STX-TE	9	0.529
	2	AN-GE-C-CF-FEP-CAZ-CRO-STX-TE-LV	10	0.588
	3	GE-S-C-AM-CF-FEP-CTX-CRO-CIP-STX-TE-LV	12	0.706
	4	GE-S-C-AM-CF-FEP-CTX-CRO-AmC-CIP-STX-TE-LV	13	0.765
	5	AN-GE-NET-S-C-CF-FEP-CRO-AmC-CIP-STX-TE-LV	13	0.765
	6	GE-S-C-AM-CF-FEP-CTX-CAZ-CRO-CIP-STX-TE-LV	13	0.765
	7	GE-S-C-AM-CF-FEP-CTX-CAZ-CRO-CIP-STX-TE-LV	13	0.765
	8	GE-S-C-AM-CF-FEP-CTX-CAZ-CRO-CIP-STX-TE-LV	13	0.765
	9	GE-S-C-AM-CF-FEP-CTX-CAZ-CRO-CIP-STX-TE-LV	13	0.765
	10	AN-GE-S-C-AM-CF-FEP-CTX-CAZ-CRO-CIP-STX-TE-LV	14	0.824
	11	AN-GE-S-C-AM-CF-CTX-CAZ-CRO-AmC-CIP-STX-TE-LV	14	0.824

AN = amikacin, GE = gentamicin, NET = netilmicin, S = streptomycin, C = chloramphenicol, AM = ampicillin, CF = cephalothin, FEP = cefepime, CTX = cefotaxime, CAZ = ceftazidime, CRO = ceftriaxone, AmC = amoxicillin–clavulanate, CIP = ciprofloxacin, STX = trimethoprim–sulfamethoxazole, TE = tetracycline, LV = levofloxacin.

**Table 3:** Antimicrobial resistance in ESBL-EC strains isolated from livestock.

Antibiotic Group	Phenotype						Genotype				
Aminoglycoside	AN	GM	NET	S			*acc(3)*-VI	*aad*A1	*str*A	*str*B	
	63.3% (19/30)	93.3% (28/30)	16.6% (5/30)	83.3% (25/30)			0% (0/30)	60.0% (18/30)	13.3% (4/30)	33.3% (10/30)	
β-lactam	AM	CF	FEP	CTX	CAZ	CRO	*bla*_CTX-M_	*bla*_CTX-M2_	*bla*_CTX-M3_	*bla*_TEM_	*bla*_SHV_
	56.6% (17/30)	73.3% (22/30)	50.0% (15/30)	73.3% (22/30)	26.6% (8/30)	70.0% (21/30)	23.3% (7/30)	0% (0/30)	0% (0/30)	33.3% (10/30)	0% (0/30)
Sulfonamide	STX						*sul*1	*sul*2	*sul*3		
	66.6% (20/30)						23.3% (7/30)	43.3% (13/30)	13.3% (4/30)		
Tetracycline	TET						*tet*A	*tet*B			
	86.6% (26/30)						73.3% (22/30)	13.3% (4/30)			
Quinolone	LVX						*qnr*A	*qnr*B			
	50% (15/30)						0% (0/30)	20% (6/30)			

## Data Availability

Data available on request.
